# Blocking negative effects of senescence in human skin fibroblasts with a plant extract

**DOI:** 10.1038/s41514-018-0023-5

**Published:** 2018-04-11

**Authors:** Ingo Lämmermann, Lucia Terlecki-Zaniewicz, Regina Weinmüllner, Markus Schosserer, Hanna Dellago, André Dargen de Matos Branco, Dominik Autheried, Benjamin Sevcnikar, Lisa Kleissl, Irina Berlin, Frédérique Morizot, Francois Lejeune, Nicola Fuzzati, Sandra Forestier, Alix Toribio, Anaïs Tromeur, Lionel Weinberg, Juan Carlos Higareda Almaraz, Marcel Scheideler, Marion Rietveld, Abdoel El Ghalbzouri, Erwin Tschachler, Florian Gruber, Johannes Grillari

**Affiliations:** 1 Christian Doppler Laboratory for Biotechnology of Skin Aging, Vienna, Austria; 2 Department of Biotechnology, University of Natural Resources and Life Sciences, Vienna, Austria; 3 Department of Biology and Women Beauty, Chanel R&T, Pantin, France; 4 Actives Department, Chanel R&T, Pantin, France; 5 Institute for Diabetes and Cancer (IDC), Helmholtz Zentrum München, German Research, Center for Environmental Health, Neuherberg, Germany; 6 Joint Heidelberg-IDC Translational Diabetes Program, Heidelberg University Hospital, Heidelberg, Germany; 7 Molecular Metabolic Control, Medical Faculty, Technical University Munich, Munich, Germany; 8 German Center for Diabetes Research (DZD), Neuherberg, Germany; 9 Department of Dermatology, Leiden University Medical Center, Leiden, The Netherlands; 10 Division for Biology and Pathobiology of the Skin, Department of Dermatology, Medical University of Vienna, Vienna, Austria

## Abstract

There is increasing evidence that senescent cells are a driving force behind many age-related pathologies and that their selective elimination increases the life- and healthspan of mice. Senescent cells negatively affect their surrounding tissue by losing their cell specific functionality and by secreting a pro-tumorigenic and pro-inflammatory mixture of growth hormones, chemokines, cytokines and proteases, termed the senescence-associated secretory phenotype (SASP). Here we identified an extract from the plant *Solidago virgaurea* subsp. *alpestris*, which exhibited weak senolytic activity, delayed the acquisition of a senescent phenotype and induced a papillary phenotype with improved functionality in human dermal fibroblasts. When administered to stress-induced premature senescent fibroblasts, this extract changed their global mRNA expression profile and particularly reduced the expression of various SASP components, thereby ameliorating the negative influence on nearby cells. Thus, the investigated plant extract represents a promising possibility to block age-related loss of tissue functionality.

## Skin aging: Plant extract blocks negative effects of senescence

A plant extract from the goldenrod (*Solidago virgaurea*) ameliorates the negative influence of senescent cells, which accumulate in the skin with aging and create a tissue environment that leads to an impaired skin structure and contributes to tumour formation. Ingo Lämmermann and a team of scientists under the lead of Johannes Grillari at the University of Natural Resources and Life Sciences, Vienna, Austria, screened several plant extracts for their potential to attenuate such detrimental effects of senescent cells on the skin. One extract from *S. virgaurea* indeed delayed the aging process of skin cells in vitro and reduced the secretion of tumour promoting molecules and pro-inflammatory factors, thereby ameliorating the negative influence on nearby cells. Thus, the investigated plant extract represents a promising possibility to block the age-associated loss of tissue functionality in human skin.

## Introduction

Cellular senescence is involved in the development of age-related diseases and the loss of tissue functionality with age. Senescent cells accumulate in vivo and their selective elimination increases the healthspan of mice.^1,2^ While transiently present senescent cells have beneficial functions in wound healing,^3^ their chronic persistence and accumulation with age negatively affects the surrounding tissue by the senescence-associated secretory phenotype (SASP). This consists of pro-inflammatory cytokines and chemokines, extracellular matrix (ECM) remodelling proteases and growth factors and results in a vicious cycle of progressive functional loss in tissues and organs.^4–6^ Senescent cells are irreversibly cell-cycle arrested via the p53–p21^CIP1^ or the p16^INK4a^–Rb axis, accumulate senescence-associated β-galactosidase activity (SA-β-gal) and display a typical morphology.^7^

One of the first organs where senescent cells have been identified in vivo, is the skin,^8^ which contains between 20%^9^ and 60%^10^ of senescent fibroblasts. The dermis of human skin can be divided into two distinct layers, the upper papillary dermis and the lower reticular dermis. Fibroblasts isolated from the papillary dermis have a lean spindle-like morphology, a higher proliferative capacity and a lower sensitivity towards contact inhibition than their flat and irregular-shaped reticular counterparts.^11^ They also differ in the production of ECM and growth factors,^11,12^ their response to growth factors and epidermal signalling^13,14^ and only papillary fibroblasts seem to be able to support the formation of a fully differentiated human skin equivalent (HSE).^15,16^ When aged in vitro or in vivo, changes in cell characteristics are almost exclusively seen in papillary fibroblasts, which led to the hypothesis that the age-related atrophy in the papillary dermis of human skin might involve a gradual de- or trans-differentiation process of the papillary to the reticular phenotype.^15,16^ We suggest to term this process “papillary to reticular transition” (PRT).

Negative effects of cellular senescence can be counteracted by: (i) delaying the loss of cell type specific functionality mediated by senescence-associated de- or trans-differentiation (like e.g. by PRT), (ii) interfering with the negative effects of SASP or by (iii) selectively eliminating senescent cells.^17^

Indeed, several clinically approved drugs including glucocorticoids, metformin, rapamycin and JAK inhibitors attenuate the SASP.^18–21^ In addition, senolytic substances have been identified including quercetin, dasatinib, navitoclax, piperlongumine, fisetin, A1331852, A1155463 and FOXO4 inhibiting peptides.^22–26^

*Solidago virgaurea*, also known as goldenrod, is traditionally used as an anti-inflammatory herbal medicine. Compounds isolated from *S. virgaurea* are reported to have cytotoxic, anti-microbial, anti-mutagenic, anti-fungal, analgesic, anti-inflammatory, anti-oxidative and diuretic activity.^27–33^ A recent study identified 3,4,5-tri-*O*-caffeoylquinic acid as the constituent with the highest reduction of tumour necrosis factor-alpha and interleuin (IL)-1β concentrations in a carrageenan-induced rat paw oedema model.^27^ However, the effect of extracts from *S. virgaurea* on cellular senescence and fibroblast subpopulations have not been studied so far.

Here we report an alcoholic extract of *Solidago alpestris* (1201) with the ability to block negative effects of senescence in human skin fibroblasts including the SASP and PRT in vitro.

## Results and discussion

### Long-term treatment with 1201 slightly delays replicative senescence and PRT of human dermal fibroblasts

**Fig. 1:**
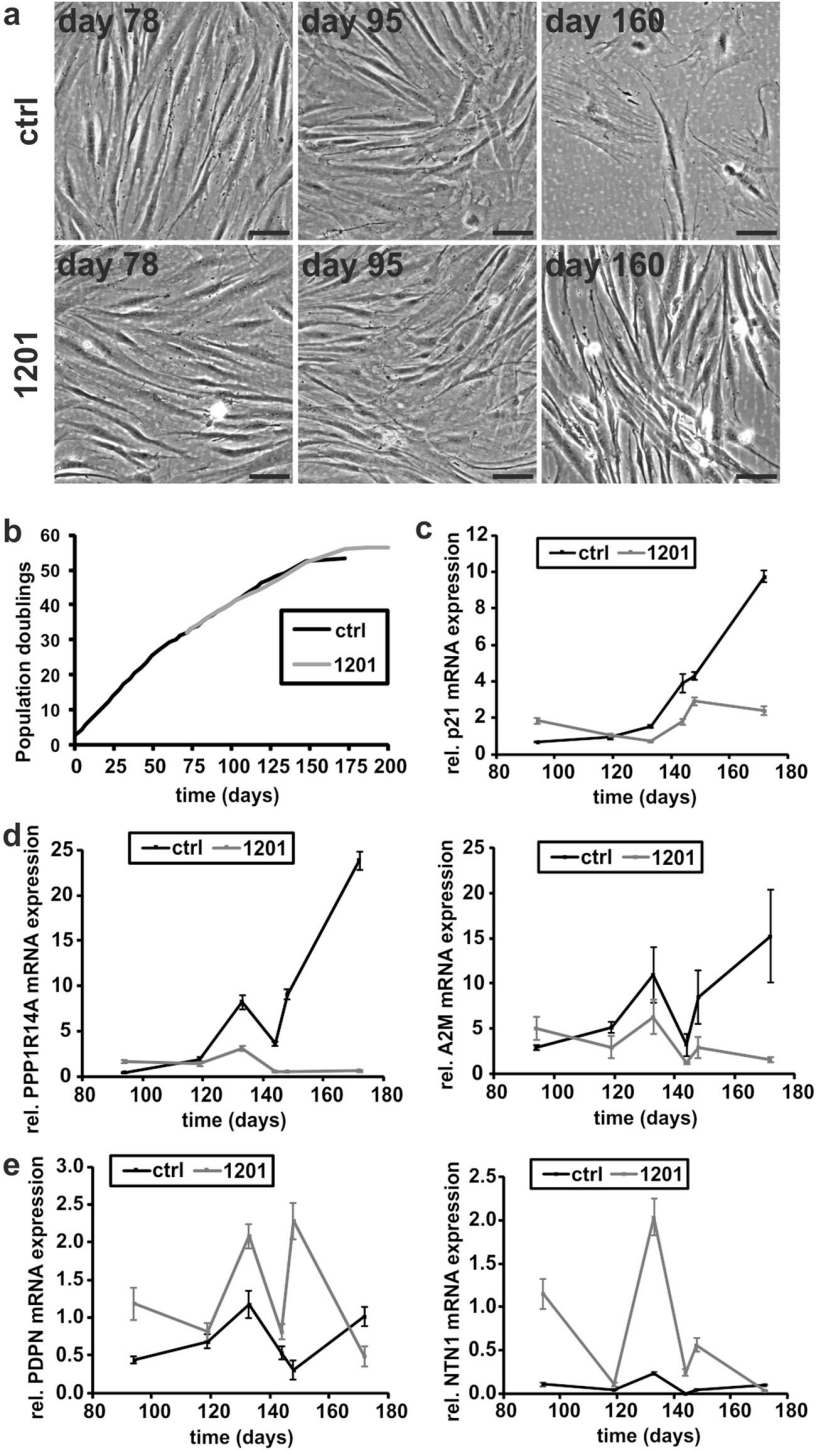
1201 delays the acquisition of a senescent phenotype. **a** HDFs were cultivated with growth medium supplemented with 1201 from day 71 (PD 32) of the replicative lifespan experiment onwards. Control cells were cultivated with normal growth medium. Microscopic pictures were taken at ×100 magnification. Scale bar, 100 µm. **b** Growth curve of the replicative lifespan experiment. At the end of the replicative lifespan the cell number was determined at regular intervals and population doublings were adjusted using the equation $\mathrm {PD}{{ = }}{\mathrm {PD}_{\mathrm {prev}}}{{ + }}\mathrm{3.32} \ast (\mathrm {log}N - {\mathrm {log}}N_{\mathrm {prev}})$ to compensate for decreasing cell density. At regular intervals during the lifespan experiment, RNA samples were prepared and the mRNA expression of **c** p21, **d** markers for the papillary and **e** reticular phenotype was determined with RT-qPCR. Expression levels of replicatively young (PD 11.5) control cells were set to 1. Data represent one experiment and error bars are calculated from four technical replicates

In order to identify plant extracts with activities that influence the PRT, we screened seven different plant extracts, one of which had to be excluded from further analysis due to a cytotoxic effect. As selection criteria for the plant extracts we focused on the ability to induce cellular characteristics of papillary human dermal fibroblasts (HDFs) and reduce the activity of SA-β-galactosidase. 1201 showed the clearest effects in terms of changing cell morphology and of reducing SA-β-galactosidase activity and was therefore selected for further studies (Supplementary Fig. S1 and S2). To investigate the consequences of long-term exposure to 1201, we cultivated HDFs from population doubling (PD) 32 onwards in the presence of 1201. 1201-treated cells retained a papillary-like morphology and cell density comparable to replicatively young cells for up to 160 days in culture, compared to 95 days for controls (Fig. 1a and Supplementary Fig. S3 and S4). While the treatment with 1201 did not have a substantial effect on growth rate or replicative lifespan (Fig. 1b), it did preserve the cellular morphology of early passage cells.

To test the hypothesis that PRT and the cellular ageing process are indeed related, we compared the expression of mRNA markers for papillary and reticular fibroblasts with a marker for cellular senescence, p21 (CDKN1A). 1201 prevented the increase of p21 and the reticular markers, protein phosphatase 1 regulatory subunit 14A (PPP1R14A) and alpha-2-macroglobulin (A2M), at the end of the replicative lifespan (Fig. 1c, d). Although the papillary markers, podoplanin (PDPN) and netrin-1 (NTN1), did not show a clear trend over the course of the replicative lifespan, their expression levels usually remained below the levels of early passage cells. In line with the observed morphology and the expression of reticular markers, the expression of the papillary markers was increased by treatment with 1201, for the majority of the time points (Fig. 1e). Only in early passage cells that have not yet acquired reticular morphology or at the very latest time point, where all cells display reticular morphology, did 1201 show no effects on PRT (Supplementary Fig. S3 and S4). Thus, we conclude that (i) in line with the results of a previous study,^16^ PRT accompanies replicative senescence leading to a more reticular status of senescent fibroblasts and (ii) that PRT is postponed by 1201 treatment.

### 1201 reverses PRT

**Fig. 2:**
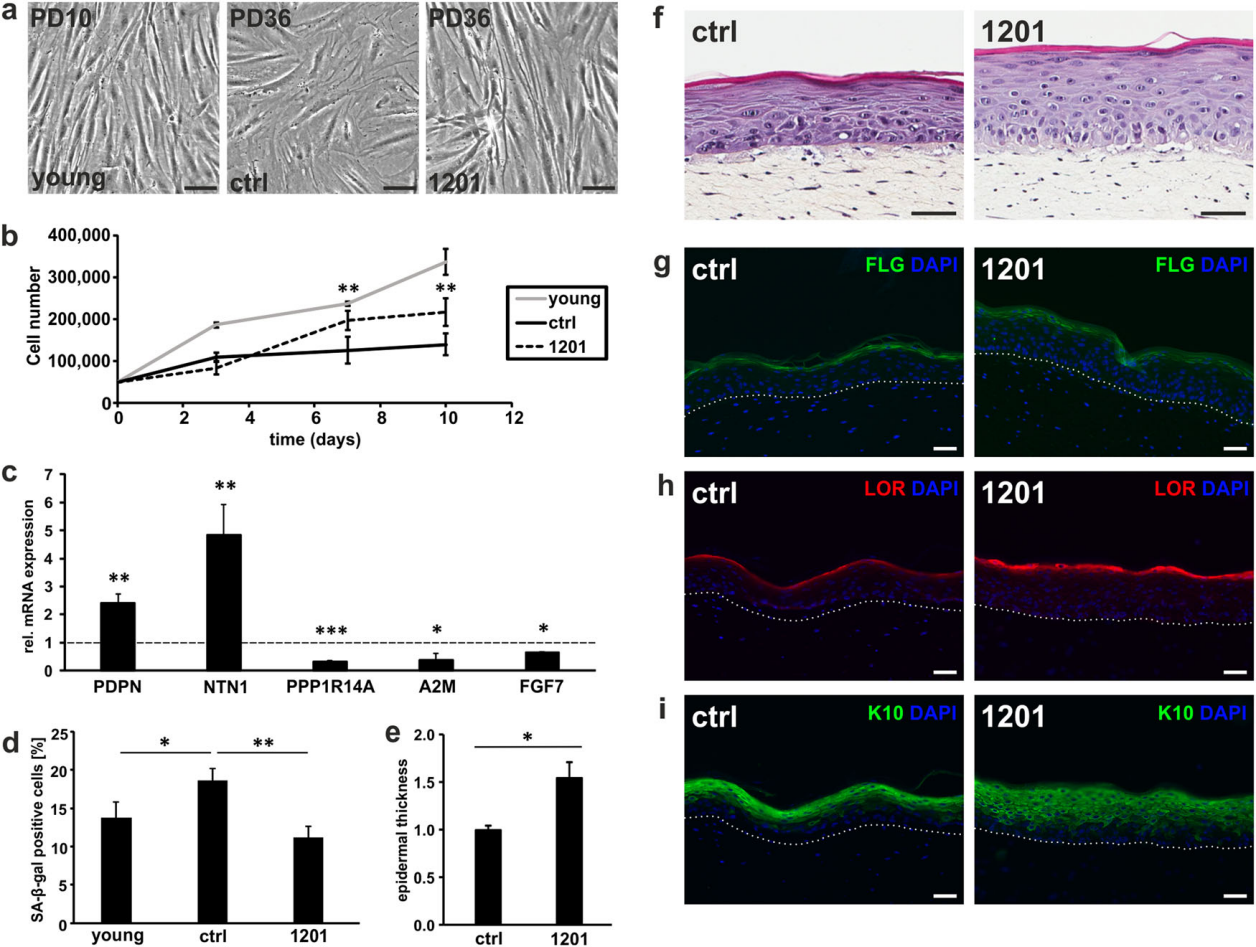
1201 reverses the papillary to reticular transition. **a** HDFs were cultivated for five PDs in growth medium supplemented with 1201. Replicatively young cells (PD 10) served as a young control. Representative pictures from one experiment at ×100 magnification. Scale bar, 100 µm. Subsequent to the treatment as described in **a**, cells were used for **b** proliferation assay, **c** RT-qPCR of markers for the papillary/reticular phenotype and FGF7 (expression levels of untreated control cells were set to 1) and **d** SA-β-gal staining. **e** Full-thickness human skin equivalents (HSE) were constructed as described in the Methods section. The average of the epidermal thickness was calculated from at least 50 measurements per HSE and the epidermal thickness of untreated HSEs was set to 1. **f** Representative pictures from one experiment as described in **e**. Scale bar, 50 µm. Immunohistochemistry of HSEs as described in **e** was performed with antibodies against **g** filaggrin, **h** loricrin and **i** keratin 10. The white dotted line separates the epidermal from the dermal layer. Scale bar, 50 µm. For **b**–**d**, data represent the average of three experiments. For **e**, data represent the average of two experiments. Statistical significance for the treatment in **b** was calculated with a two-way ANOVA (*p* = 0.004) followed by a Bonferroni post hoc test. Statistical significance for the treatment in **d** was calculated with a one-way ANOVA (*p* = 0.004) followed by a Bonferroni post hoc test

In order to confirm that 1201 reverts PRT or at least maintains the papillary phenotype, HDFs at an intermediate PD were cultivated in the presence of 1201 for 48 h and acquired a papillary-like morphology (Supplementary Fig. S5). After five PDs, this papillary-shaped young cell like morphology was maintained (Fig. 2a and Supplementary Fig. S4), in combination with higher cell densities and/or increased proliferation (Fig. 2b). In support of this, the levels of papillary markers PDPN and NTN1 were increased, while reticular markers PPP1R14A and A2M and the number of SA-β-gal-positive cells were reduced with 1201 treatment (Fig. 2c, d and Supplementary Fig. S2).

Next, we tested the effect of 1201 on the formation of full-thickness HSEs, considering that the dermal–epidermal crosstalk and the influence on the epidermal differentiation process is one of the reported differences between papillary and reticular HDFs.^11,15,16^ Indeed, when 1201 was supplemented to the medium during the differentiation process of the epidermal layer, we observed an increase in the epidermal thickness compared to untreated controls (Fig. 2e, f). In addition, 1201-treated HSEs displayed an increased extension of the stratum granulosum, as visible by increased staining for filaggrin and loricrin (Fig. 2g, h), and an increase of the suprabasal layer thickness stained with keratin 10 (Fig. 2i). A similar effect of the epidermal differentiation was previously observed when HSEs were constructed using papillary versus reticular HDFs of old donors^15^ or early versus late passage papillary HDFs^16^ as well as in organotypic cultures formed with site-matched papillary versus reticular HDFs.^11^ A reduction in filaggrin and loricrin was also observed during in vivo ageing and in HSE models for dermal and epidermal ageing.^34–36^ One cause for this phenomenon was suggested to be a paracrine effect of keratinocyte growth factor (FGF7) that was increased in reticular or in in vivo aged papillary HDFs.^11,15^ Indeed, HDFs cultivated in the presence of 1201 showed a significant reduction of FGF7 mRNA expression (Fig. 2c). Exposing HDFs to two major components of 1201, chlorogenic acid and isochlorogenic acid A, however, did not change morphology or expression of PDPN, NTN1 and PPP1R14A, suggesting that other compounds present in 1201 alone or in combination are responsible for the observed effects (Supplementary Fig. S6).

**Fig. 3:**
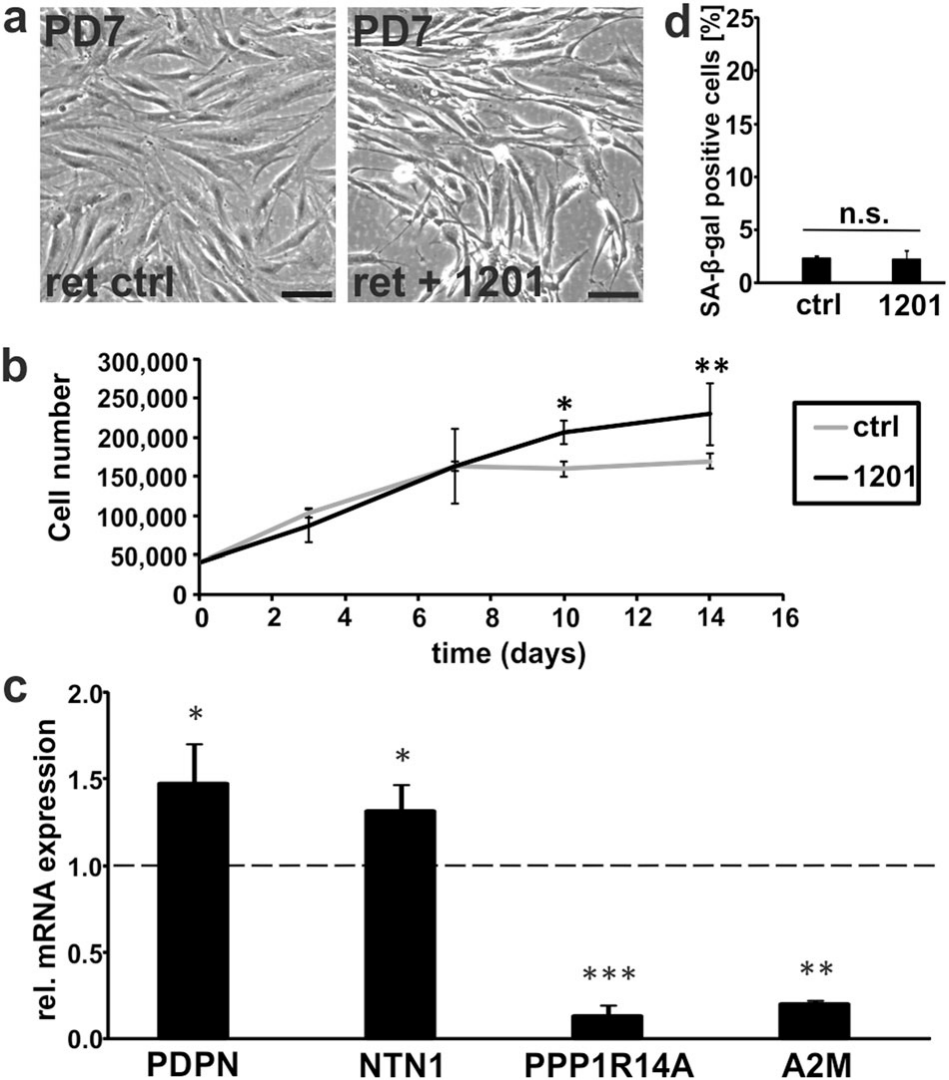
1201 induces a reticular to papillary transition in reticular HDFs. **a** Reticular HDFs were cultivated for three PDs in growth medium supplemented with 1201. Representative pictures from one experiment after cultivation for two PDs at ×100 magnification. Scale bar, 100 µm. Subsequent to the treatment as described in **a**, cells were used for **b** proliferation assay, **c** RT-qPCR of markers for the papillary/reticular phenotype (expression levels of untreated control cells were set to 1) and **d** SA-β-gal staining. For **b**–**d**, data represent the average of three experiments. Statistical significance for the treatment in **b** was calculated with a two-way ANOVA (*p* = 0.038) followed by a Bonferroni post hoc test

In addition, we isolated papillary and reticular fibroblasts from the dermis as described^37^ and confirmed mRNA marker expression (Supplementary Fig. S7). Exposure of the reticular fibroblasts to 1201 for two PDs resulted in visible morphological changes towards a papillary-like phenotype (Fig. 3a and Supplementary Fig. S4). Cells exposed to 1201 reached higher cell densities (Fig. 3b) and both papillary markers were significantly induced while the reticular markers were reduced (Fig. 3c). There was no significant change in the number of SA-β-gal-positive cells detectable (Fig. 3d and Supplementary Fig. S2), which might be due to the low basal SA-β-gal activity of early passage cells. These data together support the idea that PRT occurs with serial passaging of HDFs and is postponed by 1201.

### 1201 reduces hallmarks of senescence while not relieving cell-cycle arrest

**Fig. 4:**
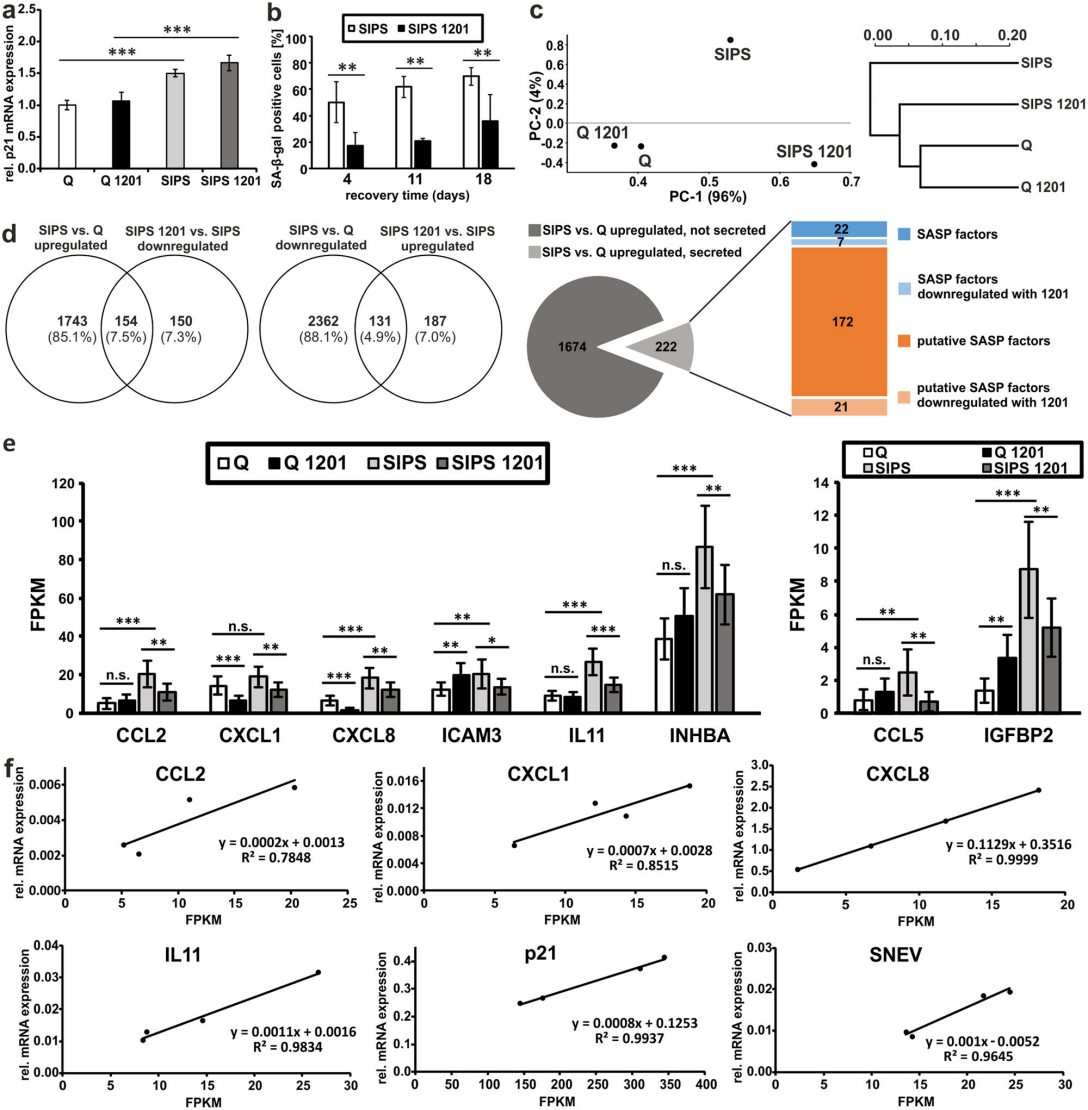
1201 attenuates the senescent phenotype in SIPS HDFs. **a** SIPS was induced by chronic oxidative stress and HDFs were subsequently cultivated for 4 days in growth medium supplemented with 1201. RT-qPCR of p21 (expression levels of untreated quiescent control cells (Q) were set to 1). **b** SA-β-gal staining of SIPS HDFs after 4, 11 and 18 days of cultivation in growth medium supplemented with 1201. Cells were treated as described in **a** and RNA-Seq was performed. **c** PCA analysis and hierarchical clustering. **d** Venn diagrams identifying the genes which are differential expressed in SIPS compared to Q and are regulated contrariwise by treatment of SIPS with 1201. The pie chart illustrates the identified SASP factors and their response to the treatment with 1201. **e** Transcript levels of all upregulated SASP factors which were regulated contrariwise by treatment with 1201, displayed as Fragments Per Kilobase of transcript per Million mapped reads (FPKM). Error bars indicate confidence intervals (95%). **f** Six genes were quantified with RT-qPCR from the same RNA samples which were used for RNA-Seq and correlated with the transcript levels derived from RNA-Seq. From **a** to **f**, data represent the average of three experiments. Statistical significance for the treatments in **a** (*p* < 0.001 for SIPS treatment and *p* = 0.088 for 1201 treatment) and **b** (*p* < 0.001) were calculated with a two-way ANOVA followed by a Bonferroni post hoc test. The *p*-values of **e** were corrected with the false discovery rate for multiple comparisons using the Benjamini–Hochberg method

To investigate the effect of 1201 on already senescent cells, HDFs were treated with 1201 after SIPS was induced with chronic oxidative stress and compared to confluent quiescent cells. While no changes on p21 were observed after 4 days of treatment with 1201 (Fig. 4a), the number of SA-β-gal-positive SIPS cells was significantly reduced already after 4 and 11 days of treatment (Fig. 4b and Supplementary Fig. S2), indicating that 1201 did also affect HDFs which were fully senescent prior to the treatment without alleviating the irreversible growth arrest.

In order to analyse the effect of 1201 on transcriptome level, RNA sequencing (RNA-Seq) was performed after four days of 1201 treatment in SIPS and quiescent control cells. Thereby, the 1201-treated SIPS cell transcriptome seems closer to that of quiescent than to SIPS cells (Fig. 4c). Raw data are publicly available at Gene Expression Omnibus (GEO) website (accession number GSE93535).

Functional annotation clustering of mRNAs that were significantly changed by SIPS and reverted back by 1201 (Fig. 4d) revealed a strong correlation with processes of the immune response among others (Supplementary Table S1). Out of 89 published SASP factors,^5,38,39^ 29 were upregulated in SIPS HDFs (Fig. 4d and Supplementary Table S1), 7 of which were significantly downregulated by 1201 (Fig. 4d, e). In addition, we included CXCL1, despite being not significantly upregulated by our SIPS treatment (*p* = 0.13), due to its role as an important chemotactic and pro-tumorigenic SASP factor and its response to the treatment with 1201. By searching the genes upregulated in SIPS HDFs for the keyword “secreted” (KW-0964), additional 193 putative SASP factors were identified, 21 of which were downregulated by 1201 on mRNA level (Fig. 4d and Supplementary Table S1). Thus, in total, out of 222 known and putative SASP factors detected, 28 were at least partially restored to normal levels by 1201 (Fig. 4d). Selected mRNAs were then confirmed by qPCRs to be differentially expressed using Pearson’s correlation, with *R*-values ranging from 0.88 to 0.99 (Fig. 4f).

**Fig. 5:**
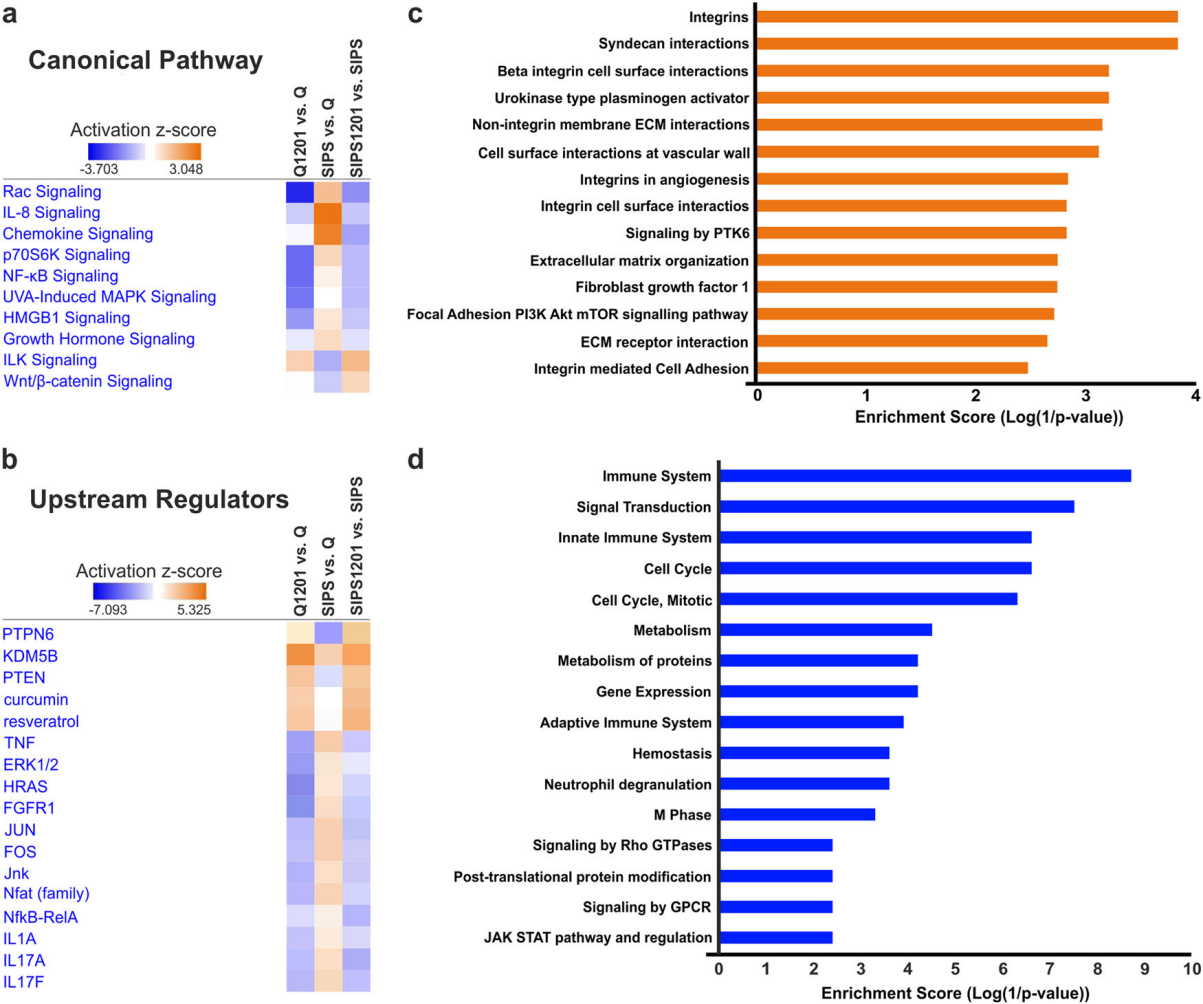
Analysis of pathways and upstream regulators affected by 1201 treatment. **a** List of selected canonical pathways and **b** upstream regulators derived from Ingenuity pathway analysis of RNA-Seq data (for complete lists refer to Supplementary Tables S2 and S3). **c** Pathway enrichment analysis of genes upregulated by 1201 in both, Q and SIPS cells, and **d** of genes downregulated by 1201 in SIPS cells. Gene expression levels were derived from RNA-Seq data. From **a** to **d**, data represent the average of three experiments

A detailed pathway analysis revealed several prominent SASP-related pathways, which were all restored by treatment with 1201 (Fig. 5a and Supplementary Table S2). In addition, ConsensusPathDB^40^ analysis revealed that 1201 downregulates pathways related to the immune system in senescent cells, including the JAK–STAT pathway (Fig. 5d).

Searching for pathways upregulated by 1201 in both, quiescent and SIPS cells, we identified pathways related to the ECM and ECM interacting proteins (Fig. 5c), which is in accordance to our data showing increased epidermal thickness in HSEs (Fig. 2e, f). Also, Wnt/β-catenin signalling, one of the few pathways activated by 1201 (Fig. 5a), was previously shown to differentially affect papillary and reticular fibroblasts in mice, in regard to their proliferation as well as their deposition of ECM during wound healing and formation of hair follicles.^13,41,42^

Predicted upstream regulators inhibited by 1201 were either related to inflammatory responses, growth factors or transcriptional regulators controlling genes involved in stress response and ageing (Fig. 5b and Supplementary Table S3). PTPN6, KDM5B and PTEN were all activated by treatment with 1201 and are factors that might mediate the observed attenuation of the SASP by potentially inhibiting the JAK/STAT, GATA4 and PI3K/AKT pathway, respectively.^43–45^ Interestingly, curcumin and resveratrol, two polyphenolic compounds with anti-tumorigenic and anti-inflammatory properties were also identified as predicted upstream regulators, indicating that the polyphenolic components of 1201 might have similar modes of action as these well-known substances.^46^

### 1201 attenuates SASP mediated effects of senescent cells

**Fig. 6:**
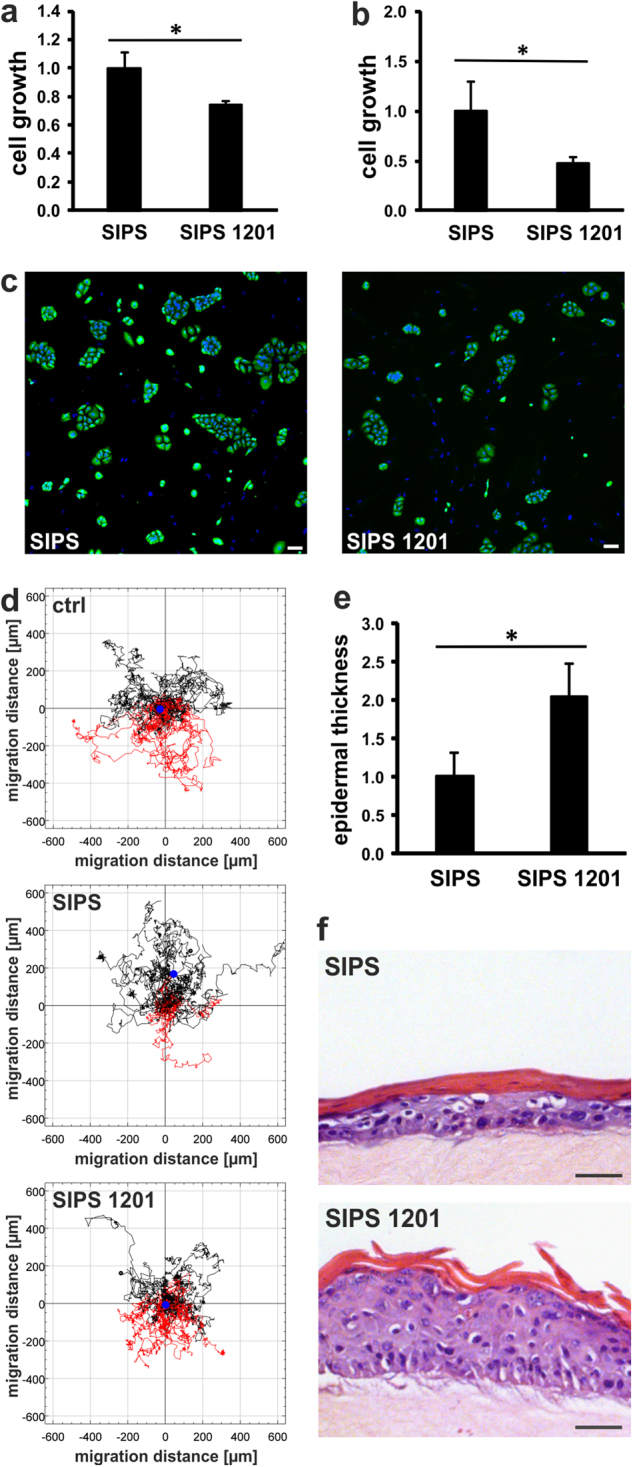
1201 blocks the effects of SIPS HDFs on other cells. SASP-attenuating properties of 1201 were evaluated by pre-treatment of SIPS HDFs with 1201 for 4 days and analysing the growth stimulation of HaCaT cells by either **a** counting K14-positive cells after co-culture with SIPS HDFs or **b** determining the cell number after cultivation with conditioned medium. **c** Representative pictures from one experiment as described in **a**. Scale bar, 100 µm. **d** Migration assay using SIPS HDF-derived conditioned medium and human PBMCs. Migratory paths are coloured in black if cells are migrating upwards, in the direction of the conditioned medium. The blue dot marks the centre of mass. **e** Full-thickness HSEs with SIPS HDFs were constructed as described in the Methods section and the epidermal thickness of control HSEs was set to 1. **f** Representative pictures from one experiment as described in **e**. Scale bar, 50 µm. For **a**, **b** and **e**, data represent the average of three experiments. For **d**, data represent one experiment

In order to see if 1201 is able to attenuate age-associated changes of intercellular communication, we tested if it decreases the previously observed pro-proliferative effect of senescent fibroblasts on the pre-neoplastic cell line HaCaT.^4^ Therefore, SIPS HDFs were pre-treated for 4 days with 1201 and subsequently HaCaT cells were either co-cultured with SIPS HDFs or cultured with conditioned medium for eight days without the addition of 1201. Indeed, SIPS HDFs pre-treated with 1201 showed a reduced growth stimulation of HaCaT cells in co-culture (Fig. 6a, c) or with conditioned medium only (Fig. 6b).

1201 also abolished chemo-attraction of SIPS HDF supernatants towards human peripheral blood mononuclear cells (PBMCs; Fig. 6d and Supplementary Fig. S8).

Finally, 1201 restored the negative impact of SIPS HDFs in HSE formation, especially on the ability of primary keratinocytes to form a fully differentiated epidermal layer, as it rescued the senescent cell dependent reduction in epidermal thickness and the impaired differentiation of keratinocytes (Fig. 6e, f), which we reported earlier (Weinmüllner et al. in preparation).

### 1201 exhibits moderate senolytic activity

In order to evaluate if 1201 also has senolytic properties, we cultivated SIPS and quiescent HDFs with 1201 for 39 days. The treatment with 1201 resulted in a significant reduction of the cell numbers of senescent cells by 30%, whereas quiescent cell numbers were not significantly affected (Supplementary Fig. S9). Treating SIPS HDFs for only 4 days with 1201 did not result in a significant reduction of the cell number, thus the attenuation of the SASP and its effect on other cells was not caused by a senolytic effect (Supplementary Figs. S9 and S10). Short-term treatment with high doses indicated that the senolytic effect of 1201 is based on the induction of apoptosis (Supplementary Fig. S11).

## Conclusion

The plant extract 1201 was not only able to delay the acquisition of a senescent phenotype, but preserved a papillary phenotype and the functionality of HDFs in regard to their ability to stimulate the formation of a full-thickness HSE. Furthermore, 1201 reverted the gene expression profile of senescent fibroblasts towards one resembling quiescent cells and reduced the expression of various SASP factors. Consequently, the negative effects of the SASP on neighbouring cells were ameliorated, namely the growth stimulation of pre-neoplastic HaCaT cells, the pro-inflammatory attraction of immune cells and the impairment of keratinocytes to differentiate and form a fully stratified epidermis. At the same time, 1201 did not override the tumour suppressive irreversible growth arrest imposed by senescence.

The identification of a treatment which simultaneously attenuates cellular senescence and induces a papillary phenotype supports the hypothesis that cellular senescence of HDFs is a differentiation process, in which the reticular phenotype represents a transition-stage on the way to the senescent phenotype. Cellular senescence of HDFs has already been proposed previously to be a (de-)differentiation process, but neither the in vivo situation nor the different dermal subpopulations have been considered so far.^47,48^

All substances, which were previously reported to ameliorate the SASP, exerted their effect by targeting NF-κB, IL1A-signalling or the JAK/STAT pathway. Pathway analysis of our RNA-Seq data provided evidence that 1201 is able to target multiple pathways involved in inflammation, ageing and tumorigenesis. The caffeoylquinic acids with their anti-inflammatory property^27^ are likely to be candidates for the SASP-attenuating property of 1201, whereas the three derivatives of quercetin, one of the three naturally occurring senolytics reported so far, could be the driving force behind the slow but significant selective elimination of around one-third of the senescent cells. The induction of a papillary phenotype might be due to interference with TGFβ-signalling which was shown to be the driving force in PRT^49^ or due to inhibition of mTOR/PI3K/AKT-signalling, which reduces SA-β-gal staining and induces a papillary-like morphology.^50^ In addition, Wnt and ILK signalling were identified as potential targets that need to be activated to counteract negative effects of senescence. However, the complete characterisation of 1201 and the identification of the molecules responsible for the identified effects will be the focus of subsequent studies.

Taken together, we have identified a plant extract that is able to block detrimental effects of cellular senescence in HDFs and to maintain their functionality. Furthermore, our results give additional evidence for a connection between cellular senescence and PRT. We have also identified numerous putative novel SASP factors, highlighting that the list of accepted SASP members might not be complete and needs to be curated in a collective effort, like e.g. by the databases available.^51^

## Materials and methods

### Plant extract (1201) preparation and characterisation

The 1201 ethanolic extract of *S. virgaurea* subspecies *alpestris* was prepared from dried aerial plant parts and solubilized with a mixture of 1,3-propanediol (Sigma-Aldrich, France) and deionised water.

Fourteen components of 1201 were identified using high-performance liquid and reverse phase chromatography followed by tandem mass spectrometry: chlorogenic acid, two isomers of caffeoylquinic acid, octulosonic acid B and C, isoquercetin, leiocarposide, rutin, quercetin arabinoside, astragalin, cynarin, 3,4- and 3,5-di-caffeoylquinic acid and nicotiflorin.

### Cell isolation

All fibroblasts and keratinocytes were isolated from skin biopsies of healthy adult donors. HaCaT cells were obtained from the German Cancer Research Center (Heidelberg, Germany). HDFs were obtained from Evercyte (Vienna, Austria). Keratinocytes and HDFs used for the construction of the HSE were isolated from a skin biopsy, which was purchased from Biopredic International (Saint-Grégoire, France). The Ethic Committee of the Medical University of Vienna (1149/2011) approved the collection of the biopsy used for the isolation of keratinocytes for the HSEs containing SIPS HDFs. Site-matched papillary and reticular HDFs were isolated from the dermis as described^37^ and their identity was confirmed by measurement of the expression levels of three papillary and three reticular mRNA markers. The site-matched papillary and reticular HDFs were isolated from surplus tissue of healthy donors by the Department of Dermatology of the Leiden University Medical Center (Leiden, The Netherlands) according to article 467 of the Dutch Law on Medical Treatment Agreement and the Code for proper Use of Human Tissue of the Dutch Federation of Biomedical Scientific Societies. The Ethic Committee of the Medical University of Vienna (1326/2013) approved the isolation of PBMCs from blood samples donated by healthy adult donors. All cell strains were tested for mycoplasma at regular intervals. The isolation of the cells was approved by the respective local ethics commission and all donors gave informed consent. Thus, this study was performed in compliance with the declaration of Helsinki.

### Cell culture

Primary human keratinocytes were cultured in DermaLife K Keratinocyte Medium (LL-0007; CellSystems). Fibroblasts and HaCaT cells were cultured with DMEM/Ham’s F-12 (1:1 mixture) (F4815, Biochrome) supplemented with 10% foetal calf serum (F7524, Sigma) and 4 mM l-glutamine (G7513; Sigma) under ambient oxygen, 7% CO_2_ and 37 °C. Cells were detached by incubation with 0.1% trypsin and 0.02% EDTA at 37 °C for 5 min and split at ratios between 1:2 and 1:6 depending on cell type and growth rate. Cells were counted using a Vi-CELL XR (Beckman Coulter) automated cell counter.

### Proliferation assay

HDFs were seeded in six-well culture plates at 40,000 cells per well. During the experiment the cells were not passaged and media was changed every 3 to 5 days. At the indicated time points the cells were detached by trypsinization and counted using a Vi-CELL XR (Beckman Coulter) automated cell counter.

### RNA isolation and RT-qPCR

Cells were lysed in TRI Reagent (Sigma) and RNA was isolated following the manufacturer’s protocol. RNA concentration and quality were measured with a ND-1000 (NanoDrop) spectrometer. cDNA was synthesised from 500 ng of total RNA using High-Capacity cDNA Reverse Transcription Kit (Applied Biosystems) and quantified with the 5x HOT FIREPol® EvaGreen® qPCR Mix Plus with ROX (Solis BioDyne) using a Rotor-Gene Q cycler (Qiagen) and the respective primer pairs (Table 1). Expression values were normalised to GAPDH mRNA.

**Table 1:** Sequences of RT-qPCR primers

Gene name	Sense primer	Anti-sense primer
*GAPDH*	CGACCACTTTGTCAAGCTCA	TGTGAGGAGGGGAGATTCAG
*PDPN*	GCATCGAGGATCTGCCAACT	CCCTTCAGCTCTTTAGGGCG
*NTN1*	TGCCATTACTGCAAGGAGGG	TTGCAGGTGATACCCGTCAC
*PPP1R14A*	GTGGAGAAGTGGATCGACGG	CCCTGGATTTTCCGGCTTCT
*A2M*	AGAGCAGCATAAAGCCCAGT	TCTCAGTGGTCTCAGTGTGGA
*p21*	GGCGGCAGACCAGCATGACAGATT	GCAGGGGGCGGCCAGGGTAT
*SNEV*	TCATTGCCCGTCTCACCAAG	GGCACAGTCTTCCCTCTCTTC
*FGF7*	TGGCAATCAAAGGGGTGGAA	GCCATAGGAAGAAAGTGGGCT
*CCL2*	GAAAGTCTCTGCCGCCCTTC	ACAGATCTCCTTGGCCACAA
*CXCL1*	TCAATCCTGCATCCCCCATAG	CAGGAACAGCCACCAGTGAG
*CXCL8*	CTCTTGGCAGCCTTCCTGATTT	ACAGAGCTCTCTTCCATCAGA
*IL11*	ATGAACTGTGTTTGCCGCCT	GGGAATCCAGGTTGTGGTCC

### SA-β-gal staining

SA-β-gal staining was performed according to standard procedures.^8^ Fifteen random images were taken per well at ×100 magnification and after randomisation positive and negative cells were counted in blinded fashion.

### Full-thickness HSE

In all, 330,000 HDF per HSE were embedded in a collagen matrix containing rat tail collagen type I (Corning), 10× DMEM (Thermofisher) and sodium bicarbonate (Gibco/Invitrogen), filled into six-well-culture inserts (Thermofisher) and placed in deep six-well culture plates (Thermofisher). After 2 h of polymerisation at 37 °C, HSEs were equilibrated in DMEM medium supplemented with 10% foetal bovine serum (Sigma) and placed at 37 °C, 5% CO_2_. After 3 days, 150,000 keratinocytes were seeded on top and submerged for 7 days in DMEM medium supplemented with 10% foetal bovine serum and growth factors (EGF, isoproterenol, hydrocortisone). Then the inserts were placed at the air–liquid interface for 7 days during which period the medium was supplemented with 1201.

HSEs were fixed in 10% formalin before embedding in paraffin and cutting into 5-µm-thick sections. Haematoxylin and eosin staining was performed using a standard protocol. Immunohistochemistry staining was performed using a standard protocol and the following primary antibodies: fillagrin (Abcam, ab17808), loricrin (Abcam, ab24722), keratin 10 (Abcam, ab9026) and the following secondary antibodies: α-mouse Alexa 488 (Abcam, ab150113) and α-rabbit Alexa 546 (Life technologies, A11010).

### SIPS

Cells were seeded at a cell density of 3500 cells/cm^2^ one day prior to the treatment. The cells were treated nine times with 100 µM H_2_O_2_ supplemented to the media for 1 h followed by a media change. Induction of premature senescence was verified by SA-β-gal staining, p21 expression, and absence of BrdU incorporation. AnnexinV/PI staining was performed to assure that the treatment was non-lethal and SIPS HDFs were cultured and monitored for over 50 days to assure that the induced growth arrest was permanent (Terlecki et al. in preparation).

### Next-generation sequencing and data analysis

Library preparation and sequencing were performed on an Illumina HighSeq 2000 Platform (GATC Biotech AG; Konstanz, Germany). All analysis steps were done according to the Tuxedo Suite Pipeline.^52^ Briefly, Illumina Casava 1.8.2 software was used for base calling. RNA-seq reads were aligned to hg19 genome assembly using TOPHAT Version 2.0.13 with default parameters.

Transcripts were assembled in Cufflinks Version 2.1.1 and differentially expressed genes were predicted by Cuffdiff. Visualisation of data was performed in R using the CummRbund package and The Unscrambler X software (Camo). Functional clustering analysis and filtering for the keyword “secreted” (KW-0964) was done using DAVID v6.8. Pathway analysis was realised with Ingenuity pathway analysis software (Qiagen). Pathway enrichment analysis was performed employing ConsensusPathDB, by using the overrepresentation analysis tool. We searched against pathways in all databases with a minimal overlap and a *p*-value cut-off of 0.0001.

### Growth stimulation of HaCaT cells in co-culture with SIPS HDF

HaCaT cells were cultivated for 48 h in KGM minimal medium (basal KGM medium supplemented only with insulin and hydrocortisone; Lonza). Then, 20,000 cells per well were seeded in six-well culture plates containing SIPS HDFs. The cells were co-cultured in KGM minimal medium for 8 days, then stained with α-K14 (ab7800; Abcam) and DAPI (Fisher Scientific). Fifteen images per condition were taken at ×50 magnification using a DMI6000 CS fluorescence microscope (Leica) and K14-positive cells were counted. SIPS 1201 HDFs were pre-treated for 4 days with 1201 prior to the experiment. During the co-culture with HaCaT cells, 1201 was not added to the medium.

### Growth stimulation of HaCaT cells with conditioned medium from SIPS HDF

Twenty thousand HaCaT cells per well were seeded in six-well culture plates and cultivated for 48 h with KGM minimal medium. Subsequently, the cells were cultivated for 8 days with conditioned medium derived from SIPS HDFs. SIPS 1201 HDFs were pre-treated for 4 days with 1201 prior to the experiment. During the conditioning of the medium and the subsequent cultivation of HaCaT cells, 1201 was not added to the medium.

### Chemotaxis assay

Chemotaxis assay with a µ-Slide Chemotaxis (IBIDI) was performed according to the manufacturer’s manual (Application Note 17 and 23), using conditioned medium from SIPS HDFs and PBMCs freshly isolated from human blood by Ficoll gradient centrifugation. Life cell imaging was performed using a DMI6000 CS microscope (Leica) equipped with a heated CO_2_ chamber (OKOLAB) and an automated stage. During 12 h, one image/2.5 min was taken. For each condition 30 cells were tracked using the manual tracking plugin and analysed with the chemotaxis and migration tool plugin (IBIDI) in ImageJ. SIPS 1201 HDFs were pre-treated for 4 days with 1201 prior to the experiment. During the conditioning of the medium and the subsequent chemotaxis assay, 1201 was not added to the medium.

### Full-thickness HSEs containing SIPS HDFs

The HSE were constructed as recently published^53^ using 250,000 SIPS HDFs and 1.5 million primary human keratinocytes per skin equivalent. SIPS 1201 HDFs were pre-treated for 4 days with 1201 prior to the experiment. During the construction of the HSE, 1201 was not added to the medium.

HSEs were fixed with Roti®-Histofix 4% (P087, Carl Roth), and paraffin-embedded for further histological analysis. Hematoxylin and eosin staining was performed using a standard protocol.

### Statistical analysis

Error bars are presented as mean ± standard deviation. If not mentioned otherwise, statistical significance was evaluated using a two-tailed Student’s *t*-test and assumption of equal variance. ANOVA was performed with SigmaPlot 12.5 and the data were tested for equal variance and normal distribution (Shapiro–Wilk). Significance levels were denoted as: *P < 0.05, **P < 0.01 and ***P < 0.001.

### Data availability

Raw data from the RNA-seq are available at the Gene Expression Omnibus with the accession code GSE93535 (https://www.ncbi.nlm.nih.gov/geo/query/acc.cgi?acc=GSE93535). All other data that support the findings of this study are available from the corresponding author upon reasonable request.

## Electronic supplementary material

Supplementary information

Supplementary Table S3

Supplementary Table S2

Supplementary Table S1

## Electronic supplementary material

**Supplementary Information** accompanies the paper on the *npj Aging and Mechanisms of Disease* website (10.1038/s41514-018-0023-5).
