# Supplementary material for: Blocking negative effects of senescence in human skin fibroblasts with a plant extract
Source: NPJ Aging Mech Dis. 2018 Apr 11;4:4. doi: 10.1038/s41514-018-0023-5 (PMC5895844; doi:10.1038/s41514-018-0023-5)
Supplement: Supplementary file 1 — Supplementary information [file 41514_2018_23_MOESM1_ESM.docx]

**SUPPLEMENTARY INFORMATION**

**
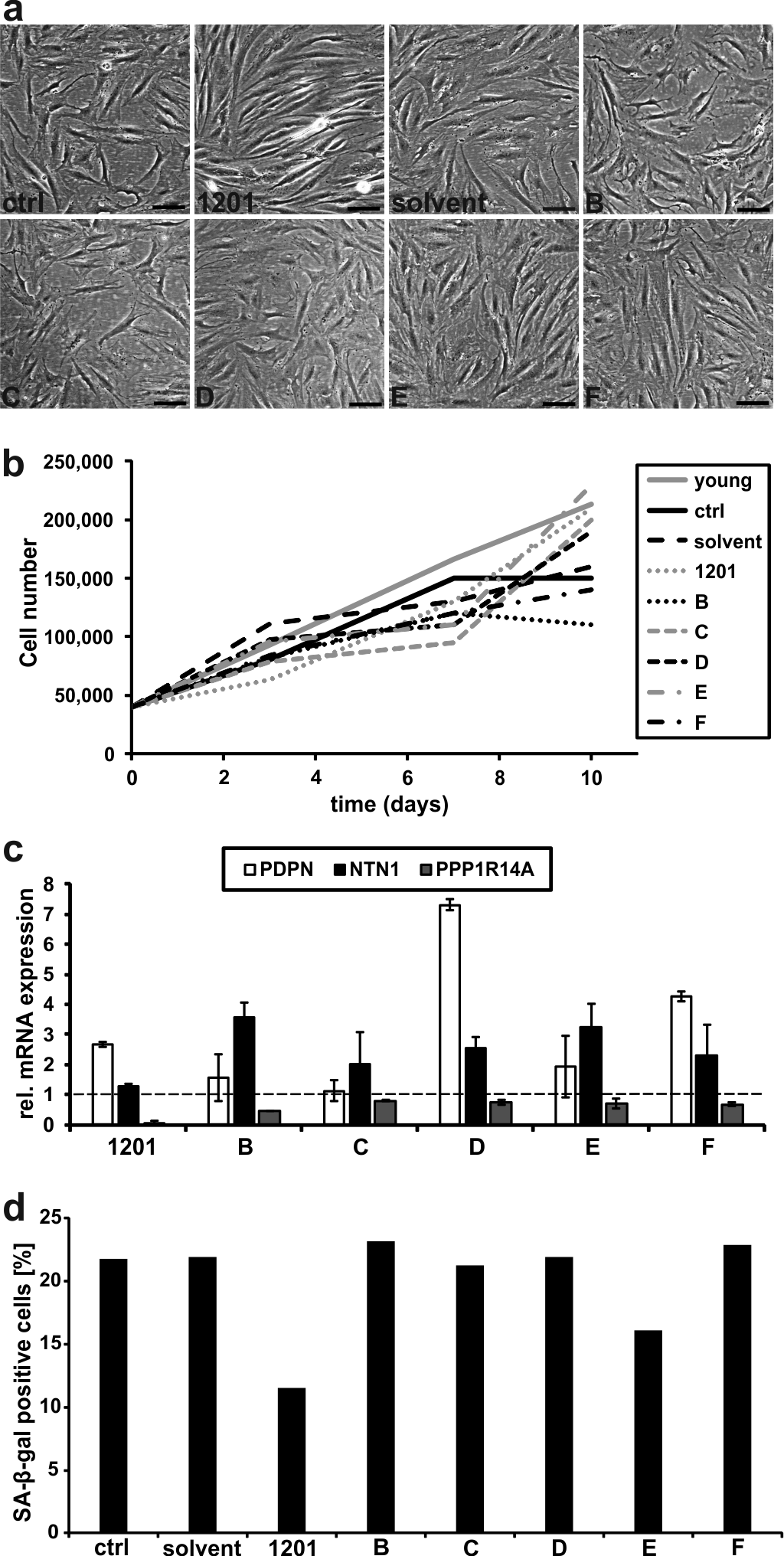
Supplementary Figure S1. Screening of several plant extracts.** (**a**) HDFs were cultivated for five PDs in growth medium supplemented with the plant extracts. Representative microscopic pictures at 100 x magnification. Scale bar, 100 µm. Subsequent to the treatment as described in **a**, cells were used for (**b**) proliferation assay, (**c**) RT-qPCR of markers for the papillary/reticular phenotype (expression levels of untreated control cells were set to 1) and (**d**) SA-β-gal staining. Data represents one experiment and for **c**, is presented as mean of 4 technical replicates.

**
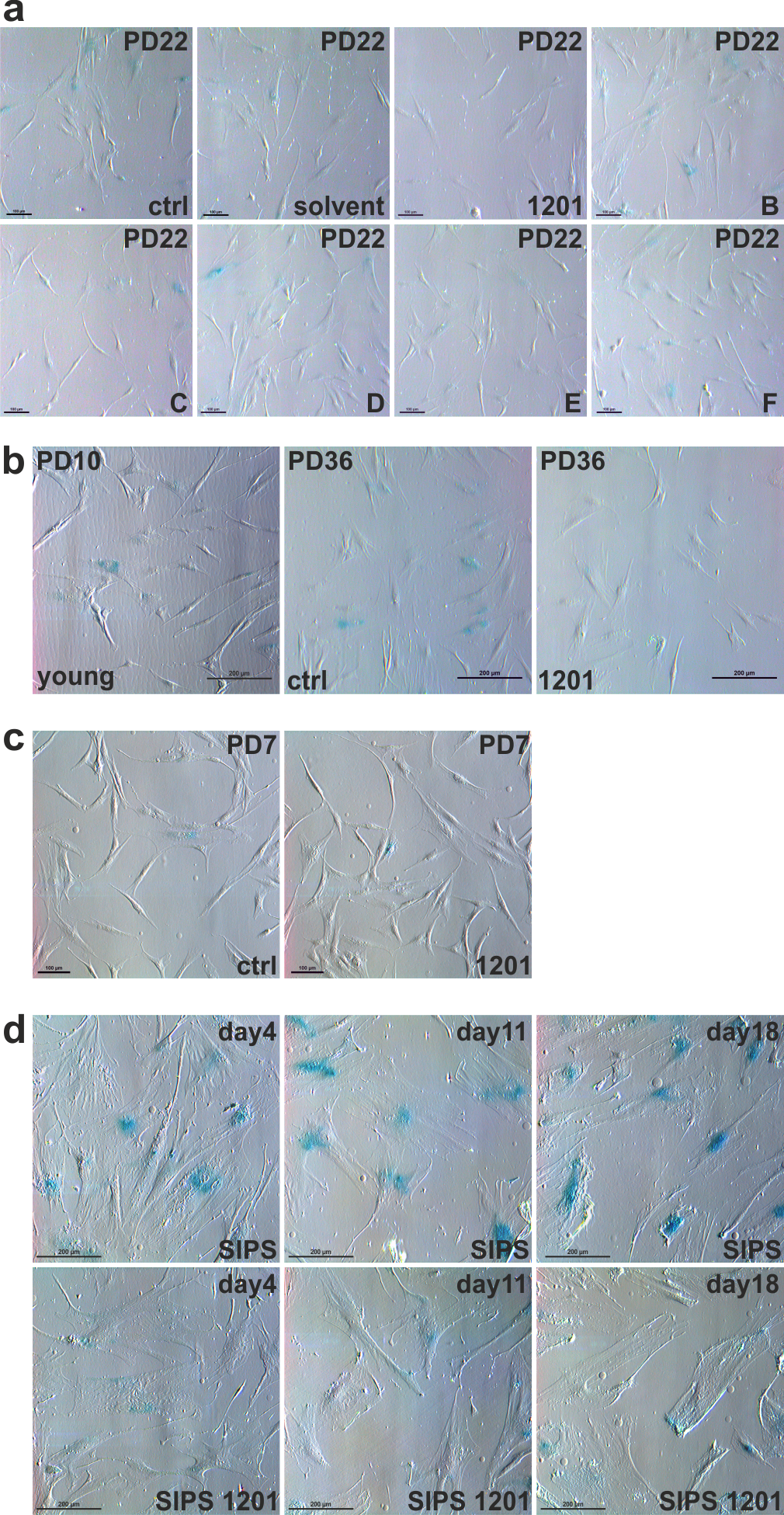
**

**Supplementary Figure S2.** **SA-β-gal stainings.** (**a**) Representative microscopic pictures of the treatment as described in **Fig. S1d** at 100 x magnification. Scale bar, 100 µm. (**b**) Representative microscopic pictures of the treatment as described in **Fig. 2d** at 100 x magnification. Scale bar, 200 µm. (**c**) Representative microscopic pictures of the treatment as described in **Fig. 3d** at 100 x magnification. Scale bar, 100 µm. (**d**) Representative microscopic pictures of the treatment as described in **Fig. 4b** at 100 x magnification. Scale bar, 200 µm.

**
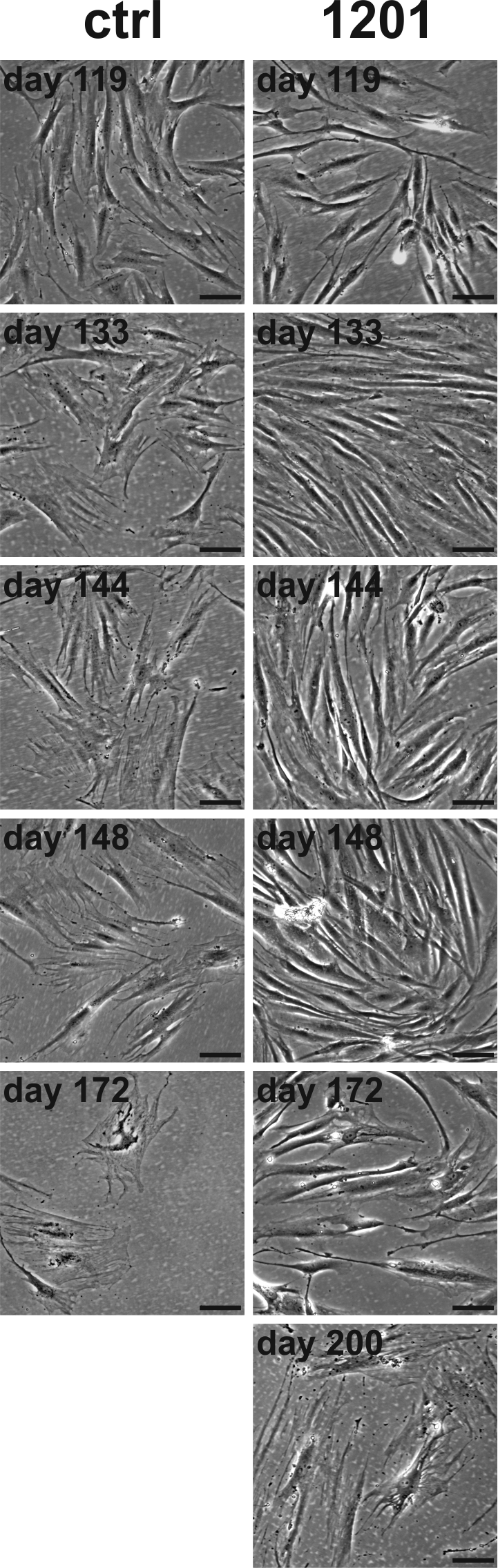
Supplementary Figure S3. 1201 helps to maintain a papillary phenotype.** Representative microscopic pictures at 100 x magnification showing cells from the replicative lifespan experiment as mentioned in **Fig. 1**. Scale bar, 100 µm.


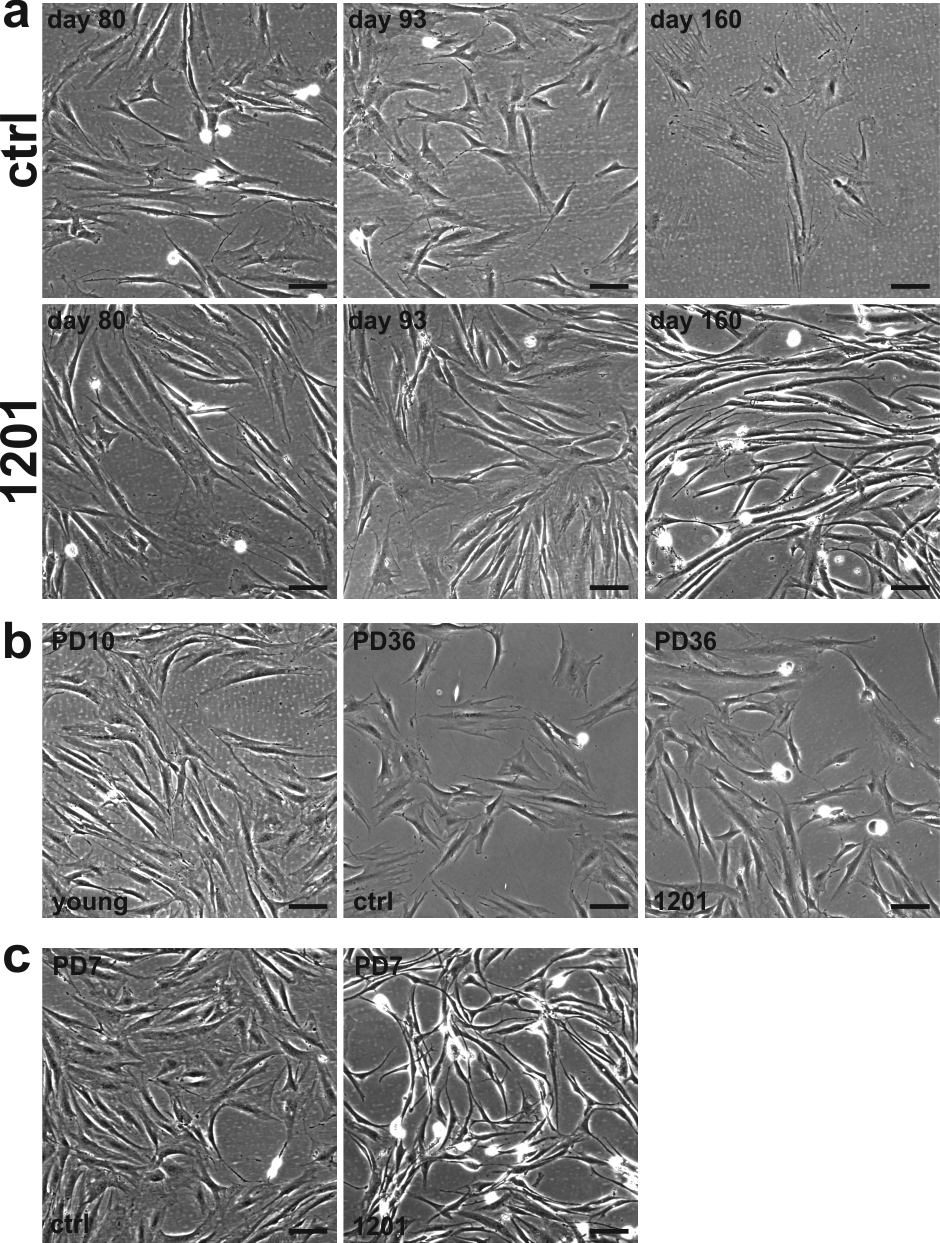
**Supplementary Figure S4.** **Representative pictures of 1201 treatments.** (**a**) Representative microscopic pictures of the treatment as described in **Fig. 1a** at 100 x magnification. Scale bar, 100 µm. (**b**) Representative microscopic pictures of the treatment as described in **Fig. 2a** at 100 x magnification. Scale bar, 200 µm. (**c**) Representative microscopic pictures of the treatment as described in **Fig. 3a** at 100 x magnification. Scale bar, 100 µm


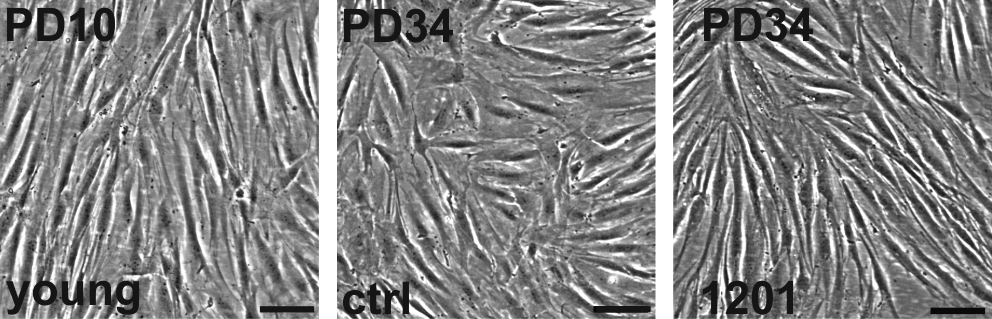


**Supplementary Figure S5. Acute treatment with 1201 induces a papillary-like morphology.** HDFs were cultivated with 1201 supplemented to the growth medium for 48 hours. Control cells were cultivated with normal growth medium. Representative pictures were taken at the end of the treatment at 100 x magnification. Scale bar, 100 µm.


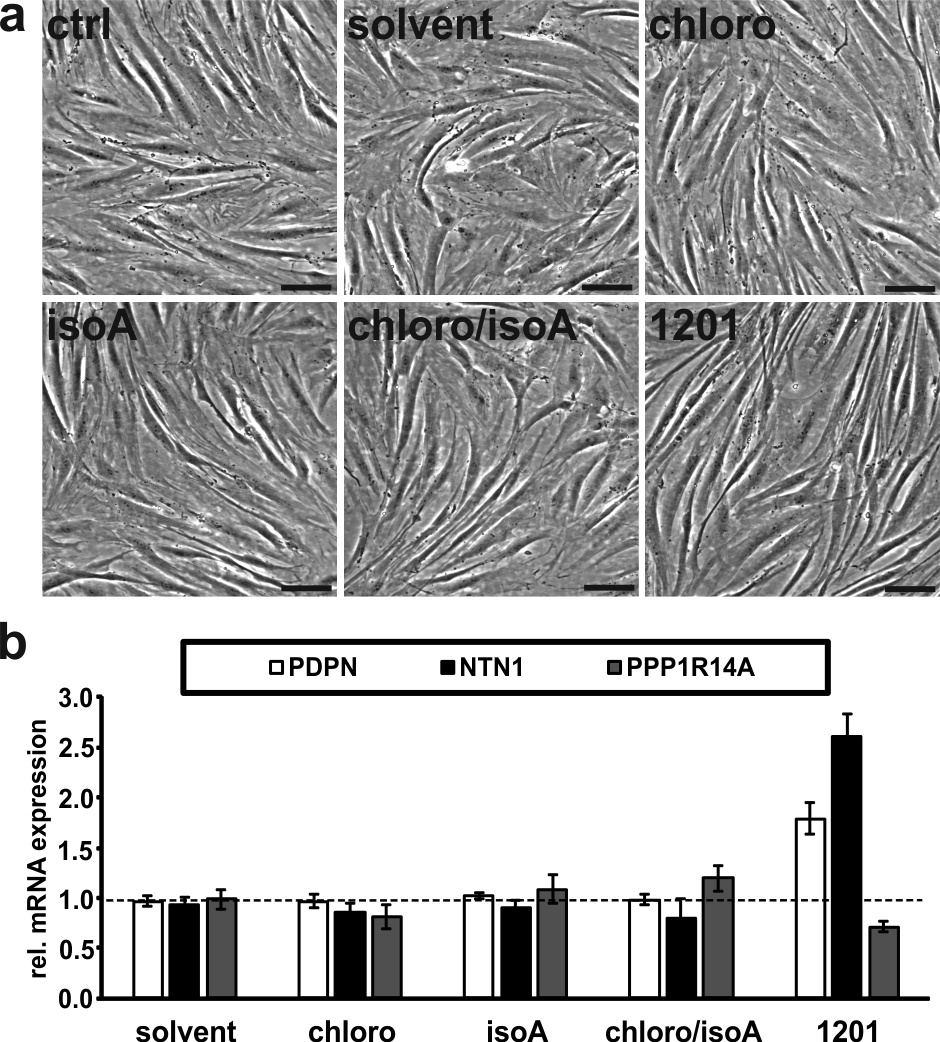
**Supplementary Figure S6. Acute treatment with 1201 and its major components.** HDFs were cultivated with 1201, 1, 3-propanediol, chlorogenic acid or isochlorogenic acid A supplemented to the growth medium for 72 hours. Control cells were cultivated with normal growth medium. Chlorogenic acid and isochlorogenic acid A were used at the concentrations at which they are present in 1201. (**a**) Microscopic pictures at 100 x magnification were taken at the end of the treatment. Scale bar, 100 µm. (**b**) Transcript levels of markers for the papillary (PDPN, NTN1) and the reticular (PPP1R14A) phenotype were analysed with RT-qPCR following the treatment as described in **a**. Expression levels of untreated control cells were set to 1 (dashed line). Expression levels of A2M were below the detection limit in all samples and are therefore not shown. Data represents one experiment and for **b**, is presented as mean of 4 technical replicates.


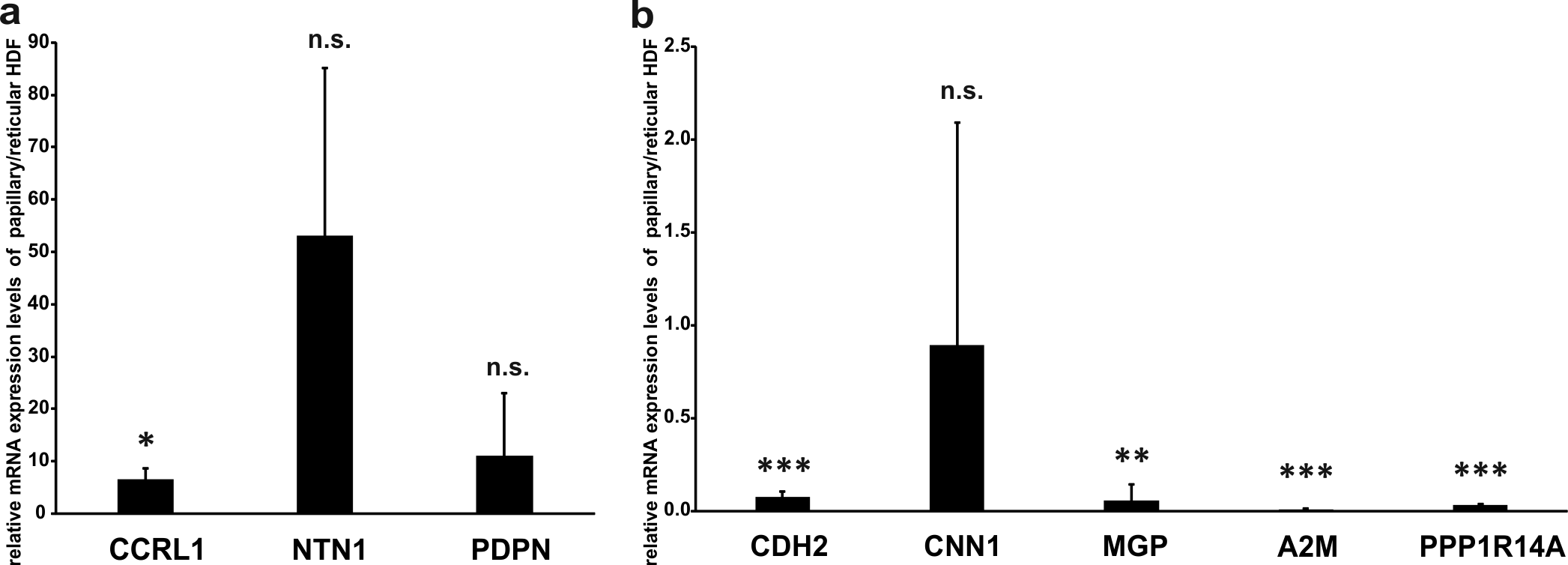


**Supplementary Figure S7. Expression of mRNA marker for papillary and reticular phenotype of site-matched papillary vs. reticular fibroblasts.** RNA samples from site-matched papillary and reticular fibroblasts of three different donors were collected and analyzed for their mRNA expression levels of markers for the papillary and reticular phenotype. Expression levels are relative to GAPDH and are depicted as ratio of papillary to reticular HDFs. Additional to the three papillary markers (CCRL1, NTN1, PDPN) and the three reticular markers (CDH2, CNN1, MGP) which are used by AEG, we tested for two reticular markers (A2M, PPP1R14A) which are used in this publication. (**a**) Relative mRNA expression levels of papillary/reticular fibroblasts for papillary markers. (**b**) Relative mRNA expression levels of papillary/reticular fibroblasts for reticular markers. Statistical significance was calculated with a one sample t-test. Data represents the average of three experiments.


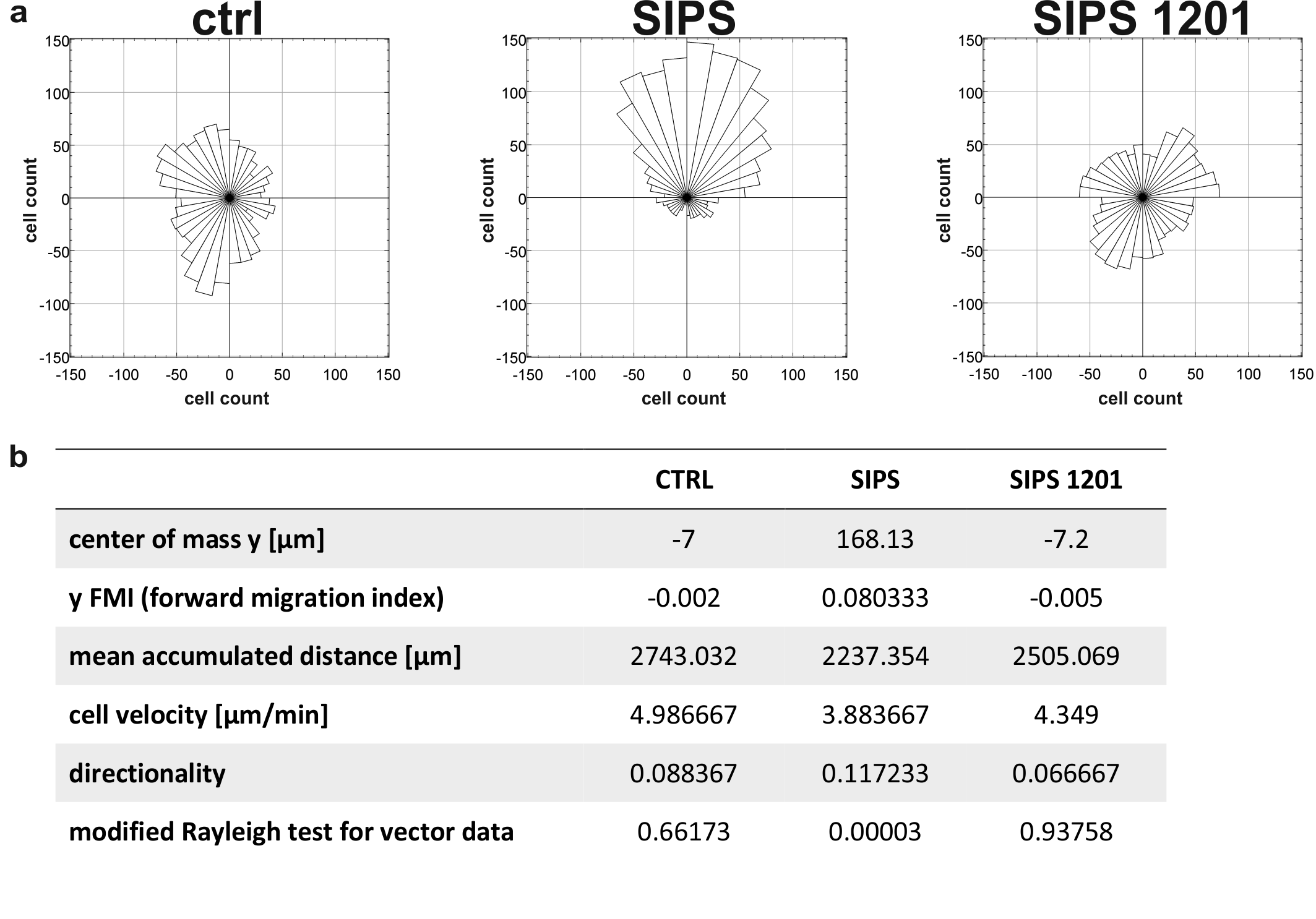
**Supplementary Figure S8. Detailed analysis of chemotaxis assay.** (**a**) Rose plots of the chemotaxis experiment as described in **Figure 6d**. Cell counts are visualized in segments, each representing 10 degrees of angle. (**b**) Statistical parameters of the chemotaxis assay.

**
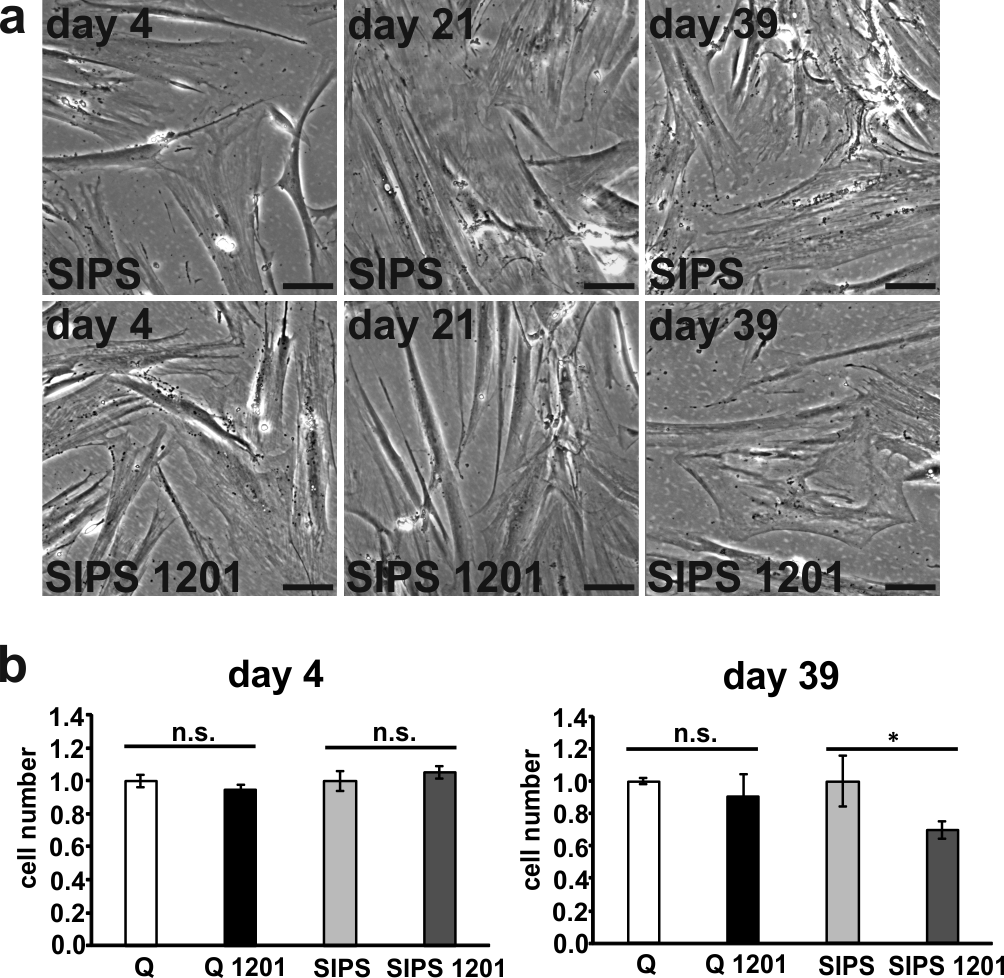
**

**Supplementary Figure S9. Long-term treatment with 1201 selectively eliminates senescent cells.** Premature senescence was induced by chronic oxidative stress in HDFs. As a control for the SIPS treatment cells were cultivated with normal growth medium, grew to confluency during the treatment and entered a quiescent state (Q). Subsequent to the SIPS treatment cells were cultivated for 39 days with 1201 supplemented to the growth medium. Control cells were cultivated with normal growth medium. (**a**) Microscopic pictures at 100 x magnification were taken throughout the course of the treatment. Scale bar, 100 µm. (**b**) At the indicated days of the treatment cells were counted and the cell number of untreated control cells was set to 1. For **a**, pictures from one representative of three experiments are shown. For **b**, data represents the average of three experiments.

**
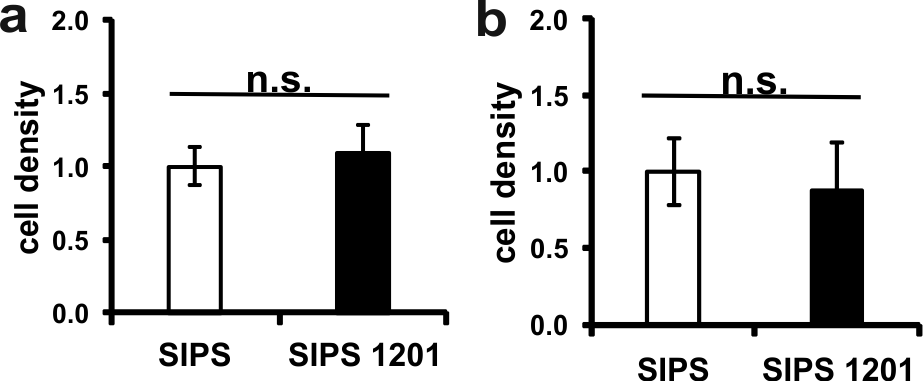
**

**Supplementary Figure S10. Short-term treatment with 1201 is not cytotoxic to senescent cells.** Premature senescence was induced by chronic oxidative stress in HDFs. Subsequent to the SIPS treatment cells were cultivated for 4 days with 1201 supplemented to the growth medium. Control cells were cultivated with normal growth medium. (**a**) HDFs were subsequently used to condition growth factor deficient keratinocyte medium for 24 hours. After collecting the conditioned medium the cells were counted and the cell number of untreated control cells was set to 1. (**b**) HDF cells were subsequently used to condition RPMI 1640 medium for 24 hours. After collecting the conditioned medium the cells were counted and the cell number of untreated control cells was set to 1. The data represents the average of three experiments.


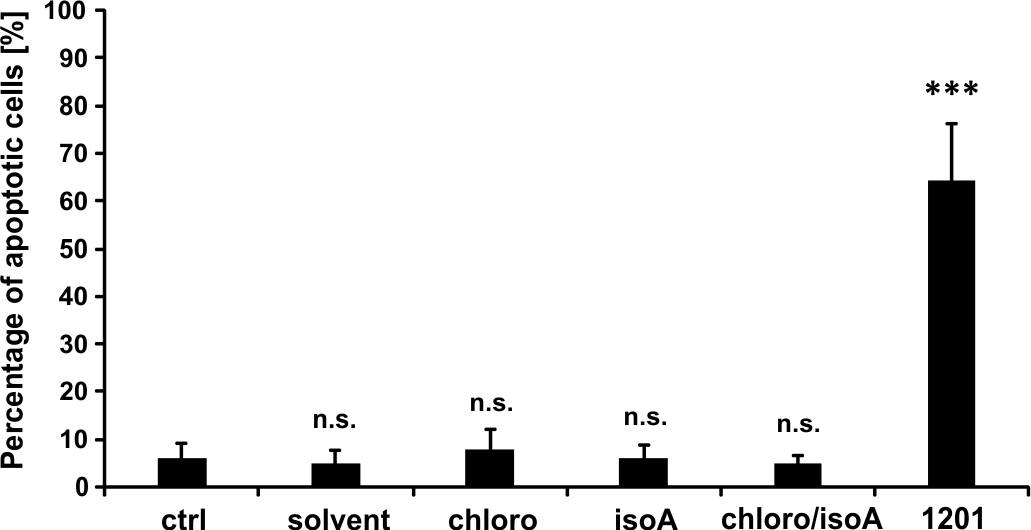


**Supplementary Figure S11. Short-term treatment with high doses of 1201 induces apoptosis.** HDFs at an intermediate PD were cultivated with 1201, 1, 3-propanediol, chlorogenic acid or isochlorogenic acid A supplemented to the growth medium for 7 days. Subsequently, the cells were stained with Propidium Iodide (PI) and an AnnexinV/Pacific blue conjugate (Thermofisher) and analysed with a flow cytometer. All cells positive for AnnexinV (early apoptotic) and double positive for AnnexinV and PI (late apoptotic) were considered apoptotic and are represented as percentage of the total analysed cell number. The data represents the average of three experiments. Statistical significance for the treatment in was calculated with a one-way ANOVA followed by a Bonferroni post hoc test.


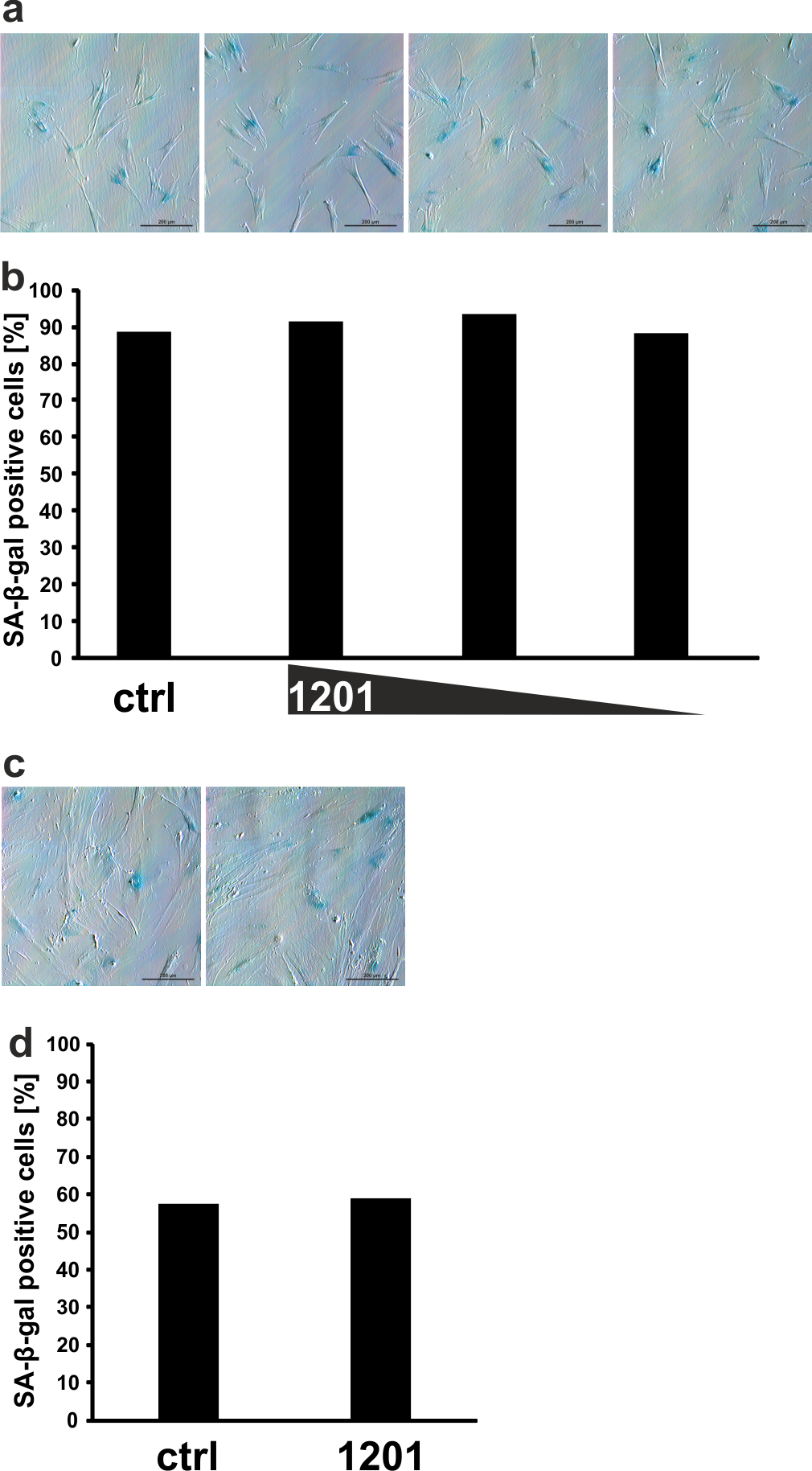


**Supplementary Figure S12. 1201 does not directly inhibit the enzymatic activity of SA-β-gal.** (**a**) Replicative senescent HDFs were treated with 1201 in different concentrations for 1 hour and subsequently stained for SA-β-gal. Representative microscopic pictures of the treatment at 100 x magnification. Scale bar, 200 µm. (**b**) Quantification of the experiment as described in **a**. (**c**) Premature senescence was induced by chronic oxidative stress in HDFs. Subsequent to the SIPS treatment cells were stained for SA-β-gal and 1201 was added to the staining solution during the procedure. Representative microscopic pictures of the treatment at 100 x magnification. Scale bar, 200 µm. (**d**) Quantification of the experiment as described in **c**. The data represents one experiment.

**Supplementary Table S1. RNA-Seq data analysis.**

**Supplementary Table S2. Complete list of canonical pathways from ingenuity pathway analysis.**

**Supplementary Table S3. Complete list of upstream regulators from ingenuity pathway analysis.**

**
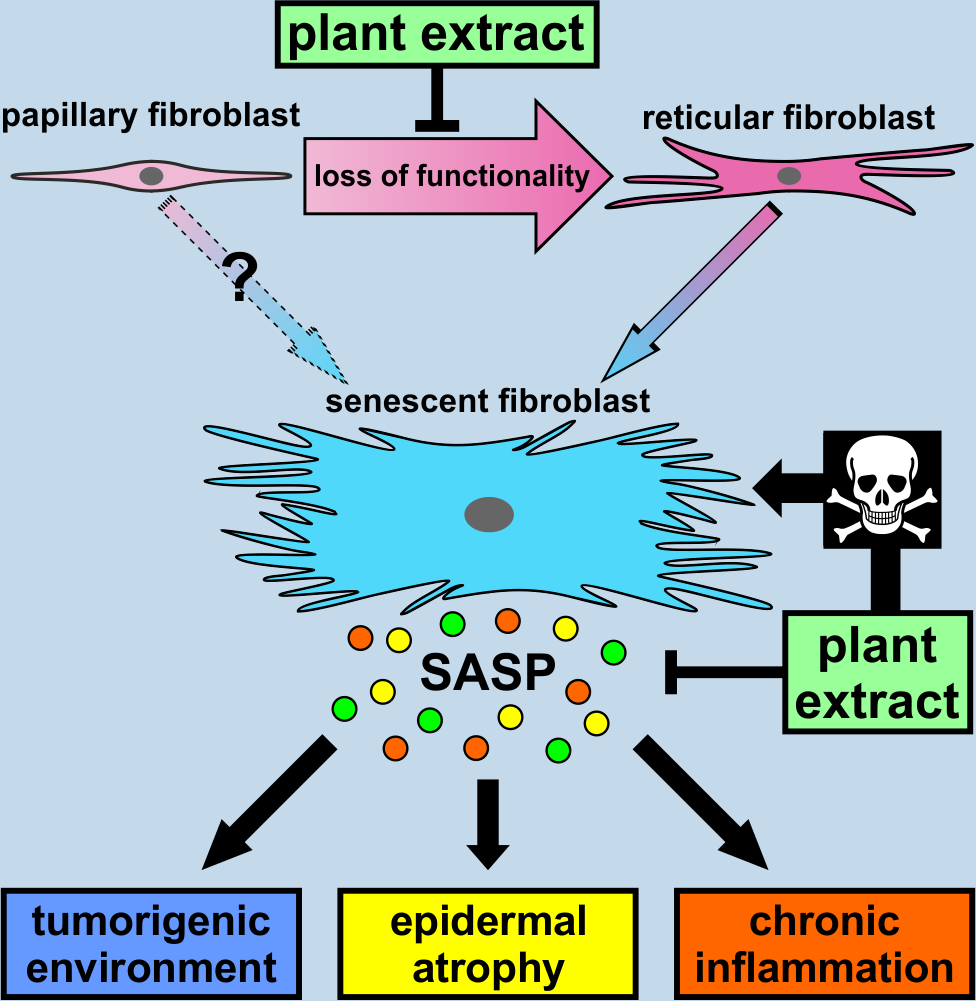
**

**Graphical abstract.**
