# Supplementary figures and images for: Blocking negative effects of senescence in human skin fibroblasts with a plant extract
Source: NPJ Aging Mech Dis. 2018 Apr 11;4:4. doi: 10.1038/s41514-018-0023-5 (PMC5895844; doi:10.1038/s41514-018-0023-5)

# Analysis Comparison 1

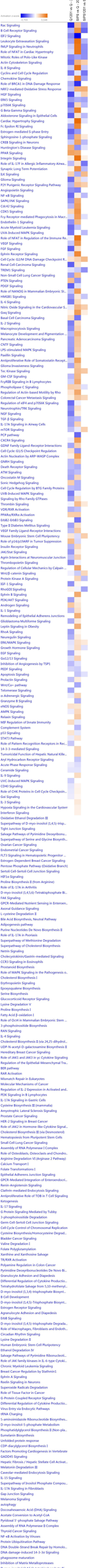

Supplement: Supplementary file 3 — Supplementary Table S2 [file 41514_2018_23_MOESM3_ESM.pdf]
